# Clinical evaluation of PET image quality as a function of acquisition time in a new TOF-PET/MR compared to TOF-PET/CT - initial results

**DOI:** 10.1186/2197-7364-2-S1-A76

**Published:** 2015-05-18

**Authors:** Konstantinos Zeimpekis, Martin Huellner, Felipe De Galiza Barbosa, Edwin Ter Voert, Helen Davison, Gaspar Delso, Patrick Veit-Haibach

**Affiliations:** Nuclear Medicine, University Hospital Zurich, Switzerland

The recently available integrated PET/MR imaging can offer significant additional advances in clinical imaging. The purpose of this study was to compare the PET performance between a PET/CT scanner and an integrated TOF-PET/MR scanner concerning image quality parameters and quantification in terms of SUV as a function of acquisition time (a surrogate of dose). Five brain and five whole body patients were included in the study. The PET/CT scan was used as a reference and the PET/MR acquisition time was consecutively adjusted, taking into account the decay between the scans in order to expose both systems to the same amount of emitted signal. The acquisition times were then retrospectively reduced to assess the performance of the PET/MRI for lower count rates. Image quality, image sharpness, artifacts and noise were evaluated. SUV measurements were taken in the liver and in white matter to compare quantification. Quantitative evaluation showed good correlation between PET/CT and PET/MR brain SUVs. Liver correlation was lower, with uptake underestimation in PET/MR, partially justified by bio-redistribution. The clinical evaluation showed that PET/MR offers higher image quality and sharpness with lower levels of noise and artefacts compared to PET/CT with reduced acquisition times for whole body scans, while for brain scans there is no significant difference. The PET-component of the TOF-PET/MR showed higher image quality compared to PET/CT as tested with reduced imaging times. However, these results account mainly for body imaging, while no significant difference were found in brain imaging. This overall higher image quality suggests that the acquisition time or injected activity can be reduced by at least 37% on the PET/MR scanner.

